# Steady Shear Rheology and Surface Activity of Polymer-Surfactant Mixtures

**DOI:** 10.3390/polym17030364

**Published:** 2025-01-29

**Authors:** Qiran Lu, Rajinder Pal

**Affiliations:** Department of Chemical Engineering, University of Waterloo, Waterloo, ON N2L 3G1, Canada; q22lu@uwaterloo.ca

**Keywords:** rheology, viscosity, surface tension, polymer, surfactant, surfactant-polymer interaction

## Abstract

Understanding the interactions between polymers and surfactants is critical for designing advanced fluid systems used in applications such as enhanced oil recovery, drilling, and chemical processing. This study examines the effects of five surfactants: two anionic (Stepanol WA-100 and Stepwet DF-95), one cationic (HTAB), one zwitterionic (Amphosol CG), and one non-ionic (Alfonic 1412-3 Ethoxylate), on the steady shear rheology and surface activity of two polymers, namely cationic hydroxyethyl cellulose based polymer (LR-400) and anionic polyacrylamide based polymer (Praestol 2540TR). The polymer-surfactant solutions behave as shear-thinning fluids and follow the power-law model. Anionic surfactants exhibit a strong effect on the rheology of cationic polymer LR-400 solution. The consistency index rises sharply with the increase in surfactant concentration. Also, the solutions become highly shear-thinning with the increase in surfactant concentration. The effects of other surfactants on the rheology of cationic polymer solution are small to modest. None of the surfactants investigated exhibit a strong influence on the rheology of anionic polymer Praestol 2540TR. Only weak to modest effects of surfactants are observed on the rheology of anionic polymers. The surface tension of the polymer-surfactant solution decreases with the increase in surfactant concentration. Zwitterionic surfactant Amphosol CG is found to be most effective in reducing the surface tension at a given concentration in ppm. This surfactant also raises the electrical conductivity of the solution to the largest extent. From the changes in slope of surface tension versus surfactant concentration plots, the approximate values of critical aggregation concentration (CAC) and polymer saturation point (PSP) are estimated.

## 1. Introduction

The oil and gas industry seeks optimization of the performance of the fluids used for hydraulic fracturing and drilling under high shear conditions [1]. The fluids, under such harsh conditions, need to meet very complicated rheological demands, sometimes interacting with other additives to attend to other operational needs like fracturing fluid pumping, recovery, and drilling fluid filtration control. Among the fracturing and drilling fluids, the polymer materials, particularly hydroxyethyl cellulose (HEC) and polyacrylamide (PAM), have received tremendous attention in recent decades due to their remarkable rheological properties, chemical stability, and multi-functionality [2,3,4,5].

Hydroxyethyl cellulose is a water-soluble cellulose derivative that has excellent thickening and rheological modification performance. It is widely used in hydraulic fracturing and drilling fluids. Under high shear conditions, HEC exhibits outstanding shear-thinning behaviors, reducing the flow resistance during pumping operations [4,5]. When shear rates are low, HEC rapidly regains its viscosity and, hence, enhances the proppant-carrying capability of fracturing fluid. Moreover, during fracturing fluid recovery, the rheological properties of HEC become very important due to the possibility of substantial viscosity reduction by chemical or enzymatic degradation, allowing efficient flowback to the surface [6,7]. HEC is widely used in drilling fluids as an effective fluid loss additive. It forms homogeneous and compact filter cakes on the wellbore wall, hence minimizing the penetration of fluids into the formation. This property maintains wellbore stability as well as minimizing formation damage [8,9].

The second very important class of water-soluble polymers is polyacrylamides, which find widespread applications in hydraulic fracturing and drilling fluids. Its high molecular weight and flexible molecular structure allow PAM to present excellent shear-thinning properties, enabling it to tolerate high flow velocities during pumping fracturing fluid and circulation of the drilling fluid [10,11]. In addition, PAM has superior viscosity and viscoelasticity, allowing it to support the reliable transport of proppants, especially in deep fractures. In the recovery after fracturing fluid injection, the chemical degradability of PAM ensures rapid decline in viscosity, allowing it to flow back efficiently [12]. Finally, PAM serves as an effective fluid loss additive in drilling fluids, with good compatibility with the formation pore to seal fractures and voids for well fluid loss control [13,14].

In practice, surfactants are also added to hydraulic fracturing and drilling fluids to enhance the performance of polymers. Surfactants improve the properties of polymer-based fluid formulations under high shear conditions, mainly by altering interfacial tension and molecular interactions. In fracturing fluids, surfactants reduce the viscosity of the fluid during pumping by causing partial disruption of polymer chain entanglements. The reduction in viscosity reduces frictional flow resistance and decreases energy consumption [15,16]. The surfactants also enhance the shear stability of the fluid by forming hydrated layers around polymer molecules and, therefore, prevent overstretching or breaking of the polymer chains under high shear stress [17]. Moreover, surfactants reduce rapid shear-thinning behavior because of the strengthening of intermolecular interactions, which can help retain viscosity during high-shear operations with consequent proper proppant transport to the fracture zone [18].

In drilling fluids, surfactants help in the optimum development of filter cake properties and in the improvement of the fluid loss additives’ performances. In addition, they enhance the suspension efficiency of solid particles such as weighting agents or fluid loss materials, thus assuring a homogeneous distribution of solids within the fluid [19]. This prevents sedimentation, hence uniform deposition of filter cakes on the wellbore wall, improving the sealing of formations with high permeability [20].

The synergistic interactions between polymers, for example, HEC and PAM, together with surfactants in water, are generally dominated by electrostatic, hydrophobic, and steric interactions, which together will affect the rheological and interfacial properties of a fluid. These mechanisms alter the molecular configuration of the polymers, enhance fluid performance in high shear conditions, and provide additional structural stability [21,22]. A brief description of electrostatic, hydrophobic, and steric interactions is as follows:

(a) Electrostatic Interactions:

Electrostatic interactions may result from the interaction of ionic surfactants with oppositely charged sites on the polymer chains. For example, anionic surfactants may bind to positively charged groups on a polymer to induce chain contraction by electrostatic attraction. The charge neutralization of polymer chains could also enhance chain entanglements and the formation of network of polymer chains. Conversely, electrostatic repulsion upon like charges may lead to an expansion of the polymer chain. The dynamic interaction of this kind will affect the size of the polymer coil and change the fluid viscosity. Such effects are even more pronounced in charged systems like PAM with partial hydrolysis, when carboxylate groups interact with ionic surfactants [21,23,24].

(b) Hydrophobic Interactions:

The interactions between hydrophobic species take place by association of the nonionic or amphiphilic surfactants with hydrophobic regions of the polymer chains. For example, hydrophobic tails of surfactants may associate themselves with less polar regions of the polymer to form micelle-like structures or hydrophobic domains in the fluid. Such an association could result in the enhancement of the viscoelasticity of the system, improved resistance of the system against shear degradation, and stability of the fluid under extreme conditions [25,26,27].

(c) Steric Interactions:

Surfactants physically adsorb on the surface of the polymer to create a stabilizing layer, impeding excessive entanglement of the chains under high shear due to steric hindrance. This consequently minimizes the possibility of chain breaks and enables the polymer, even under harsh stress conditions, to retain its functional properties related to viscosity and elasticity, among others [28,29,30].

The interactions just described have different origins but are complementary in their effects. The charge-based interactions are basically governed by electrostatic forces, while hydrophobic interactions influence the structural association of nonpolar segments. Steric interactions provide mechanical stabilization to the polymer-surfactant complex. All these allow the fluid to maintain optimum viscosity during fracturing or drilling operations, enhance proppant transport, and improve filtration control, ultimately contributing to the efficiency in oil and gas field applications [21,31,32,33].

Despite the importance of the problem from a practical point of view, the interactions between surfactants and polymers and their effect on rheology are still not well understood. The main objective of this work is to investigate the steady shear rheology and surface activity of polymer-surfactant mixtures experimentally, keeping in mind the final application involving formulation and design of hydraulic fracturing and drilling fluids. To that end, this paper explores the rheological properties of cationic HEC (quaternary ammonium salt of HEC) and anionic PAM under varying shear conditions and discusses the influence of five different surfactants (two anionic, one cationic, one zwitterionic, and one non-ionic) on the rheological properties and surface activity of polymer-surfactant mixtures. The experimental results presented are new. To our knowledge, the interactions between between the polymers and surfactants and their effect on rheology and surface activity selected in this work have not been studied in the literature to any significant extent.

## 2. Materials and Methods

### 2.1. Materials

**Figure 1:**
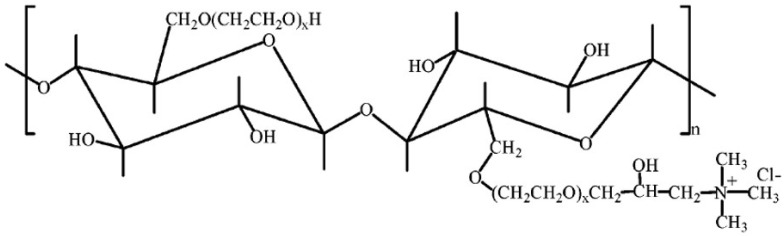
The chemical structure of LR-400.

The hydroxyethyl cellulose (HEC) used in this work was the UCARE Polymer LR-400 (herein referred to as LR-400), a cationic quaternary ammonium salt of hydroxyethyl cellulose of high purity and good water solubility. It is manufactured by Dow Chemical Co., Midland, MI, USA. LR-400 has a wide range of applications in the cosmetic and personal care fields and many other industries. Its chemical structure is presented in Figure 1 [34].

**Figure 2:**
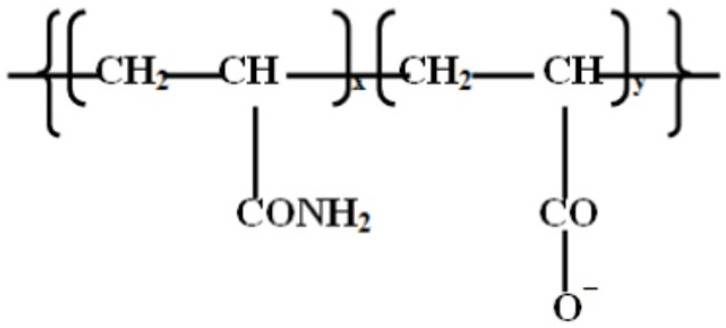
The chemical structure of Praestol 2540TR.

The PAM used was Praestol 2540TR, a purified anionic polyacrylamide soluble in aqueous solutions. Praestol 2540TR is widely used industrially as a flocculating agent and thickener in paper production, textile, food processing, and petrochemical and mineral processing industries [35]. Its chemical structure is presented in Figure 2 [36].

Five different surfactants were investigated, as listed in Table 1 below.

### 2.2. Preparation of Solutions

For the experiments, two types of solutions were prepared. One was a pure polymer solution. To prepare these solutions, LR-400 was dissolved at a concentration that varied over the range of 500 to 5000 ppm to study its rheological behavior with changing concentrations. In contrast, the Praestol 2540TR concentration was maintained at a fixed 500 ppm.

The second set of solutions consisted of mixed surfactant-polymer solutions. In these solutions, LR-400 and Praestol 2540TR concentrations were fixed at 5000 ppm and 500 ppm, respectively, to isolate and investigate the effects of surfactants. The concentration of each surfactant varied in such a way that its effect on the interaction with polymers could be systematically studied.

All solutions were prepared by adding the additives, polymer, or surfactants, slowly to deionized water with gentle stirring to ensure that complete dissolution was achieved for homogeneous solutions. All solutions were prepared at an ambient temperature.

### 2.3. Rheological Measurements

The rheological properties of the solutions were studied by means of a Fann 35A/SR 12 coaxial cylinder viscometer (Fann Instrument Company, Houston, TX, USA). In such a viscometer configuration, an inner cylinder, the so-called bob, remains fixed while the outer cylinder, so-called rotor, rotates at set rates. The inner cylinder had a radius of 1.7245 cm, while the outer cylinder radius was 1.8415 cm. The height of the bob was 3.8 cm, with the gap between the rotor and the bob, within which the fluid was to be sheared, being equal to 0.117 cm.

The viscometer recorded a plot of shear stress vs. shear rate at constant room temperature of 22 °C. Calculations of shear rate at the bob surface were based on measured rotor speed in radians per second. The relationship between shear stress and dial (torque) reading was obtained by calibration using viscosity standards of known viscosities.

### 2.4. Surface Tension Measurements

The surface tension of the solutions was measured at room temperature using a smartphone-based tensiometer and the ADSA (Axisymmetric Drop Shape Analysis) method. The instrument was supplied by Droplet Lab, Markham, ON, Canada. A pendant droplet was generated at the tip of a needle or capillary and was illuminated from the back by a light source. The pendant droplet was imaged at high resolution using a smartphone camera and analyzed using specialized software. From the calculated drop geometry, the software was able to calculate the surface tension of each solution numerically.

The measurement was performed 12 times on each solution to guarantee precision and reproducibility; thus, an average value was calculated. These very consistent measurements ensured that the experimental method was reliable.

### 2.5. Electrical Conductivity Measurements

The electrical conductivity of polymer-surfactant solutions was measured by a Thermo Orion 3 Star conductivity meter (Thermo Fischer Scientific Inc., Beverly, MA, USA). The measurements were conducted at room temperature 22 °C.

## 3. Results and Discussion

### 3.1. Rheological Behavior of Polymer Solutions

#### 3.1.1. LR-400 Polymer Solutions

**Figure 3:**
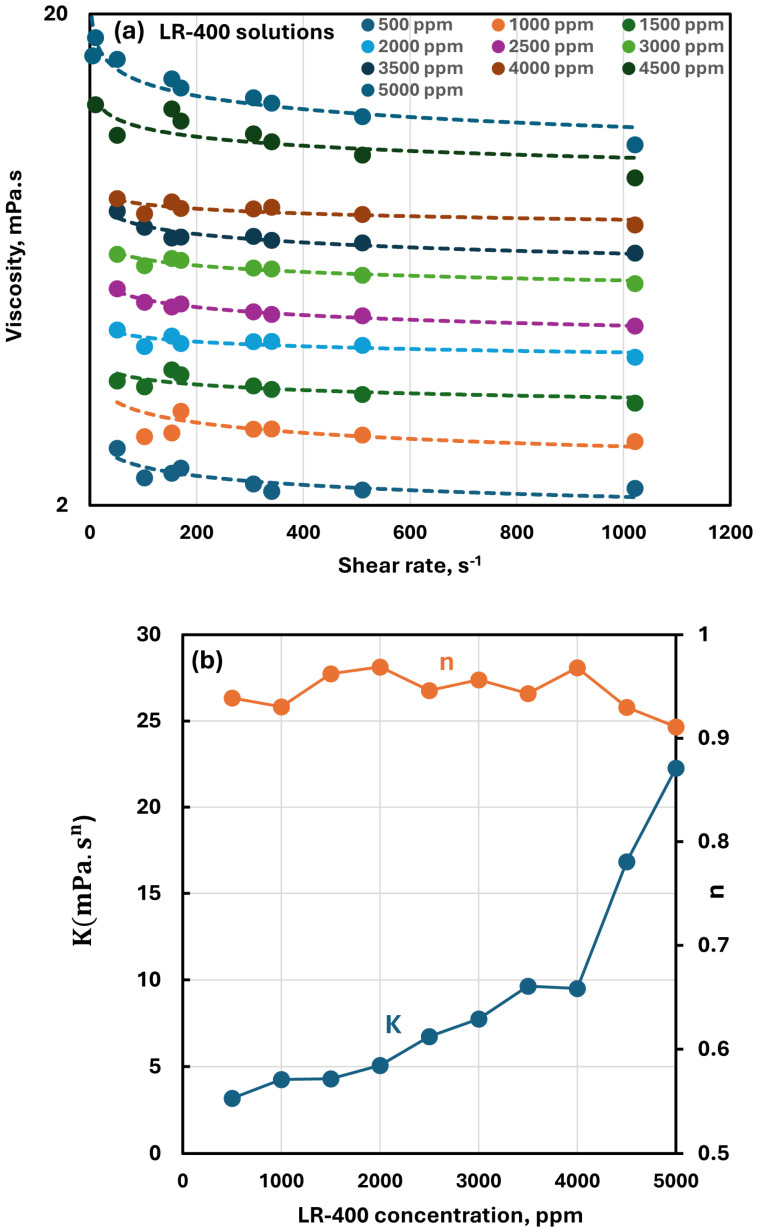
Rheological behavior of cationic LR-400 polymer solutions. (**a**) Viscosity versus shear rate. (**b**) Power-law parameters.

Viscosity versus shear rate data for cationic LR-400 solutions at different polymer concentrations are shown in Figure 3a. All the solutions show shear-thinning behavior with viscosity decreasing with the increase in shear rate due to the polymer chains orientation in the shear flow direction and hence reduction in their internal resistance. However, the degree of shear-thinning in LR-400 solutions is not large. Interestingly, the shear stress-shear rate curves, when plotted on a logarithmic scale, were straight lines, indicating that the polymer solutions follow the power-law model:

(1) $$\tau =K{\dot{\gamma }}^{n}$$

(2) $$\mu =\tau /\dot{\gamma }=K{\dot{\gamma }}^{n-1}$$

where *τ* is the shear stress, $\dot{\gamma }$ is the shear rate, K is the consistency index, n is the flow behavior index, and μ is viscosity. The power-law model is used extensively in the literature [38,39] to describe the shear stress (or viscosity) versus shear rate behavior of shear-thinning non-Newtonian fluids, including polymer solutions. Figure 3b shows the plots of power-law parameters K and n as functions of polymer (LR-400) concentration. While the flow behavior index n does not show a strong dependence on polymer concentration, the consistency index K rises sharply with the increase in polymer concentration. Also note that n is only slightly less than unity, indicating a small degree of shear-thinning. The large increase in K can be attributed to the formation of more robust intermolecular interactions and network structures as polymer chains become more densely packed at high concentrations.

#### 3.1.2. Praestol 2540TR Polymer Solutions

**Figure 4:**
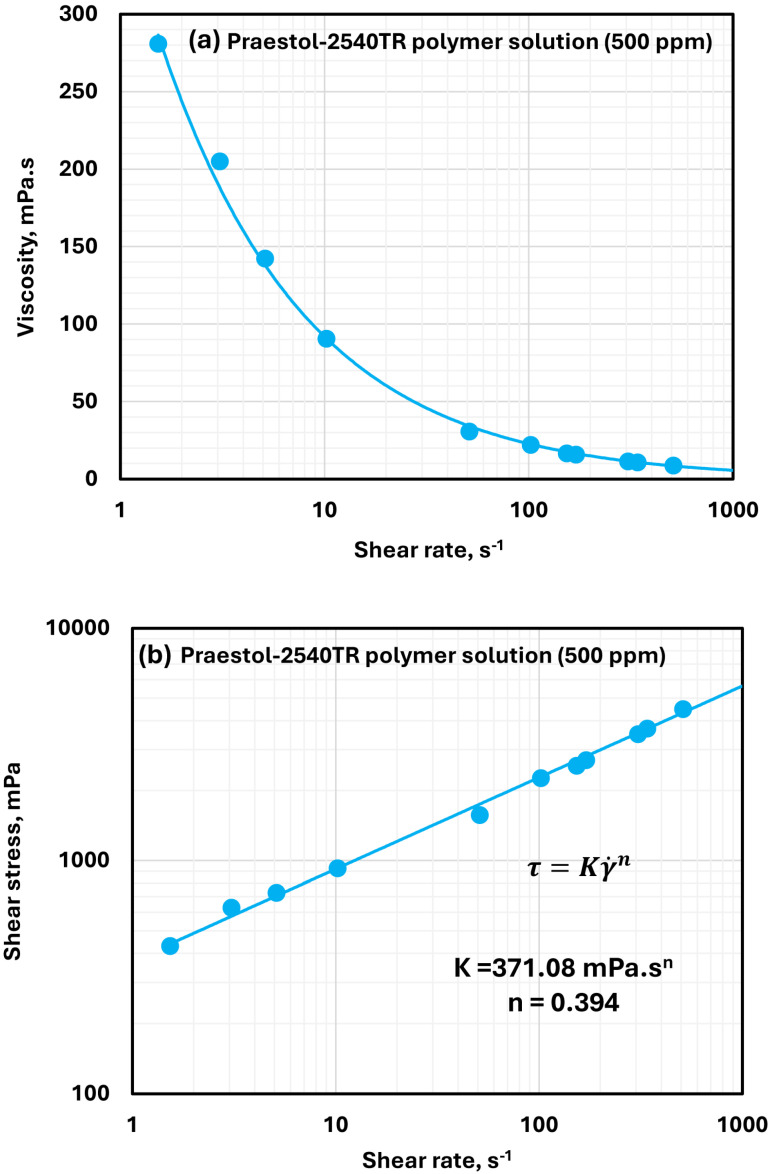
Rheological behavior of anionic Praestol 2540TR polymer solutions. (**a**) Viscosity versus shear rate. (**b**) shear stress versus shear rate.

Figure 4 displays the rheological behavior of anionic Praestol 2540TR solution at a fixed polymer concentration of 500 ppm. The plot of viscosity against shear rate, as depicted in Figure 4a, indicates that this solution is highly shear-thinning. The shear stress-shear rate relationship presented in Figure 4b follows the power-law model, with a consistency index of 371.08 *m*$Pa\cdot s^{n}$ and a flow behavior index of 0.394. The relatively low value of n underlines that the solution is highly shear-thinning in nature due to polymer chains aligning in the shear direction and causing a reduction in internal resistance. The shear-thinning behavior in polymer solutions is desirable in industrial applications such as in hydraulic fracturing and drilling since high viscosity at low shear rates suspends particles well and low viscosity at high shear rates minimizes pumping resistance.

### 3.2. Rheological Behavior of Polymer-Surfactant Solutions

#### 3.2.1. Anionic Surfactant (Stepanol WA-100) + Polymer Solutions

**Figure 5:**
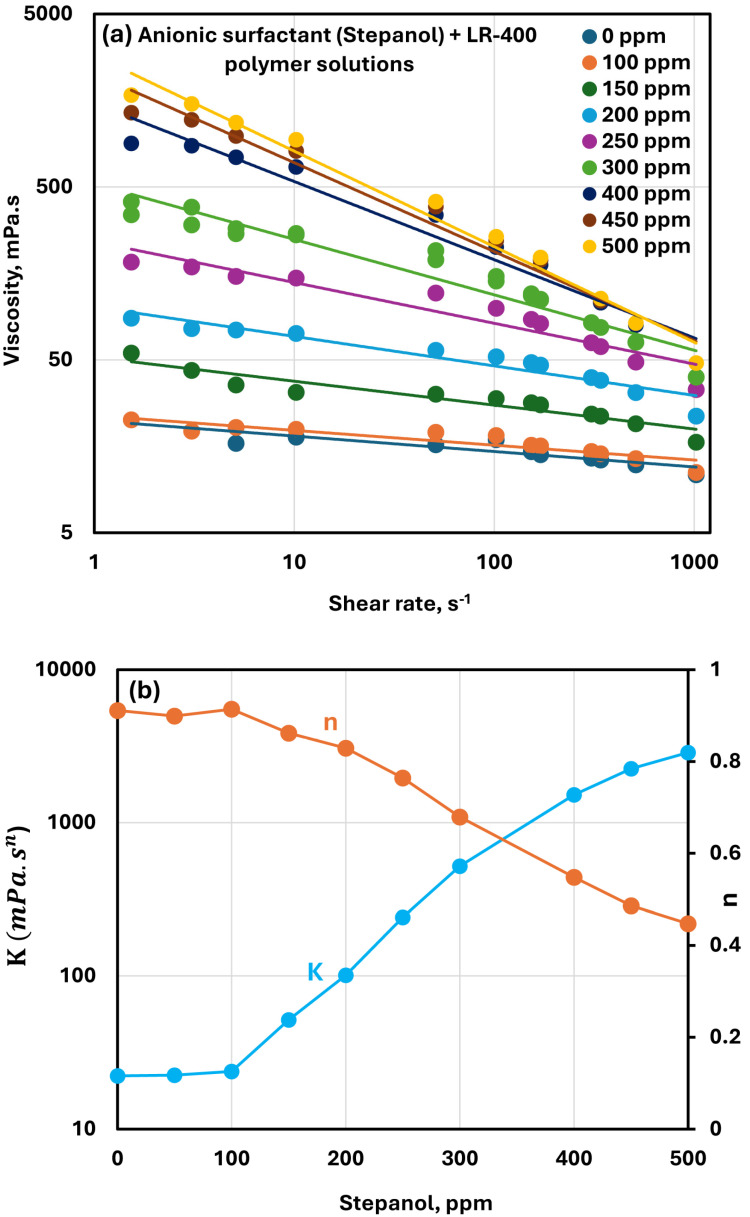
Rheological behavior of anionic surfactant Stepanol + cationic LR-400 polymer solutions. (**a**) Viscosity versus shear rate. (**b**) Power-law parameters.

The effect of the anionic surfactant Stepanol on the viscosity of cationic LR-400 polymer solution at a fixed polymer concentration of 5000 ppm is shown in Figure 5a. The data are plotted as viscosity versus shear rate. The polymer-surfactant solutions exhibit shear-thinning behavior, where viscosity decreases with increasing shear rate, and they conform to the power-law model, Equations (1) and (2), for non-Newtonian fluids. Interestingly, the viscosity of the LR-400 solution increases progressively with rising surfactant concentration, indicating enhanced structural interactions between the cationic polymer and the anionic surfactant.

The variations in the consistency index (K) and flow behavior index (n) with surfactant concentrations are depicted in Figure 5b. Up to a surfactant concentration of 100 ppm, K remains nearly constant, suggesting minimal changes in the solution structure at lower surfactant levels. Beyond 100 ppm, K increases significantly, indicating the formation of strong polymer-surfactant complexes that reinforce the network structure and boost solution viscosity. The flow behavior index (n) stays relatively stable at lower surfactant concentrations (up to 100 ppm) but decreases as the surfactant concentration rises further, signifying more pronounced shear-thinning behavior at higher concentrations. This behavior reflects strong polymer-surfactant interactions at elevated surfactant concentrations, resulting in a more entangled network structure.

**Figure 6:**
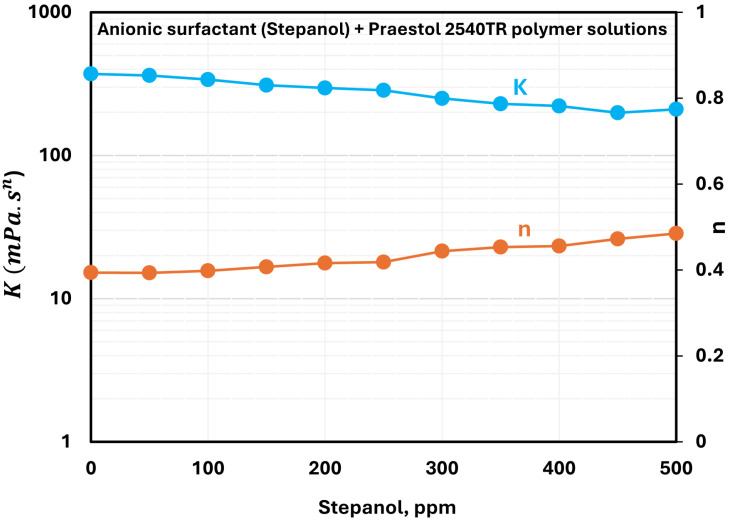
Variations of power-law parameters of anionic surfactant Stepanol + anionic Praestol 2540TR polymer solutions with surfactant concentration.

For anionic Praestol 2540TR polymer solutions, the addition of anionic surfactant Stepanol results in only a small change in the rheological properties. The changes in the consistency index (K) and flow behavior index (n) with the addition of Stepanol to Praestol 2540TR solution are shown in Figure 6. As surfactant concentration increases, n rises slightly, indicating a small reduction in shear-thinning tendency. The consistency index K also decreases only marginally. Thus, only weak interactions between anionic polymer and anionic surfactant occur, likely due to electrostatic repulsion. Nevertheless, all solutions retain their shear-thinning characteristics, consistent with the inherent behavior of Praestol 2540TR polymer solution.

#### 3.2.2. Anionic Surfactant (Stepwet DF-95) + Polymer Solutions

**Figure 7:**
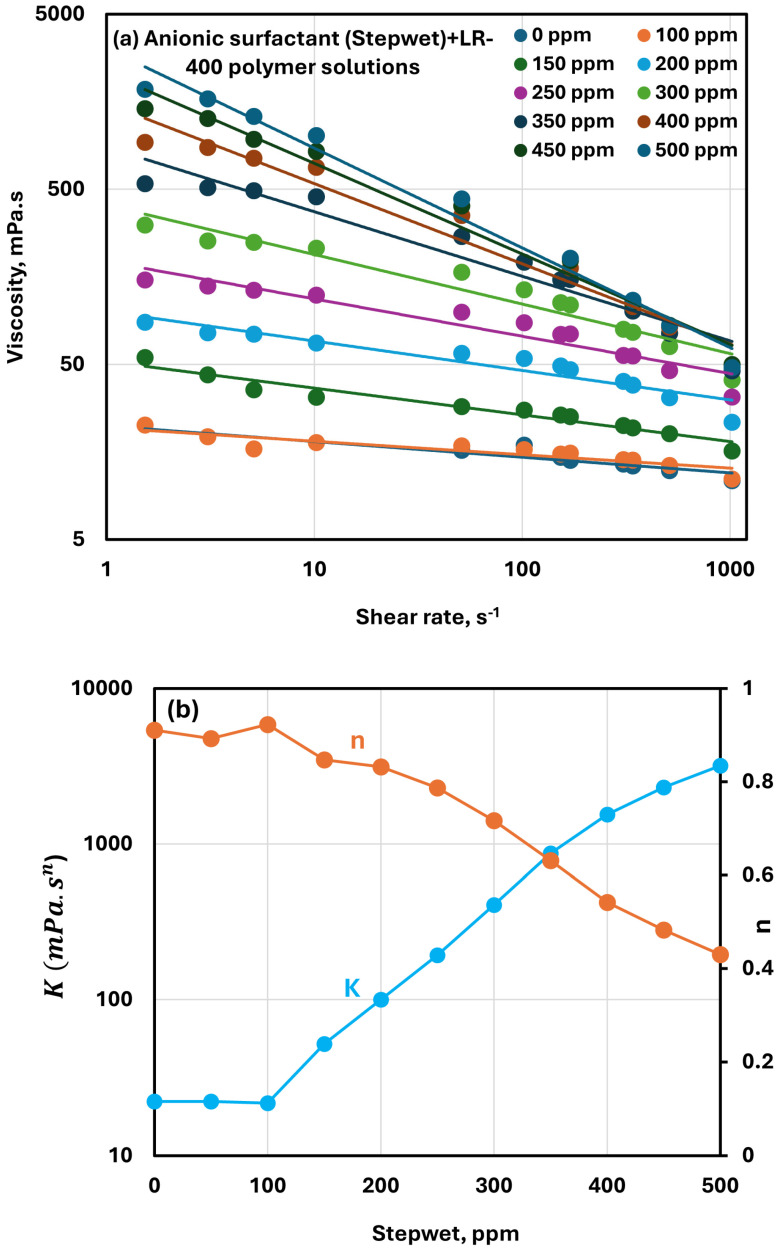
Rheological behavior of anionic surfactant Stepwet + cationic LR-400 polymer solutions. (**a**) Viscosity versus shear rate. (**b**) Power-law parameters.

The impact of the anionic surfactant Stepwet on the viscous behavior of cationic LR-400 solutions at a fixed polymer concentration of 5000 ppm is shown in Figure 7a. Viscosity increases consistently with the addition of Stepwet across all shear rates, indicating a significant interaction between the cationic polymer and the anionic surfactant. This behavior suggests the formation of polymer-surfactant complexes, which enhance the overall network structure within the solution. All solutions maintain shear-thinning characteristics, meaning viscosity decreases as shear rate increases due to the alignment of polymer-surfactant complexes along the shear flow direction.

The viscosity versus shear rate plots are linear, indicating the validity of the power-law model. The changes in the consistency index (K) and flow behavior index (n) with increasing surfactant concentration are illustrated in Figure 7b. As the surfactant concentration rises, the flow behavior index n decreases substantially, indicating an increase in shear-thinning behavior. The consistency index K also rises sharply when the surfactant concentration is increased beyond 100 ppm, reflecting a significant rise in viscosity at low shear rates. These trends suggest that the addition of Stepwet DF-95 strengthens the entanglement between polymer chains and surfactant molecules, forming a more cohesive structure. The marked changes in K and n clearly demonstrate robust interactions between the oppositely charged polymer and surfactant.

**Figure 8:**
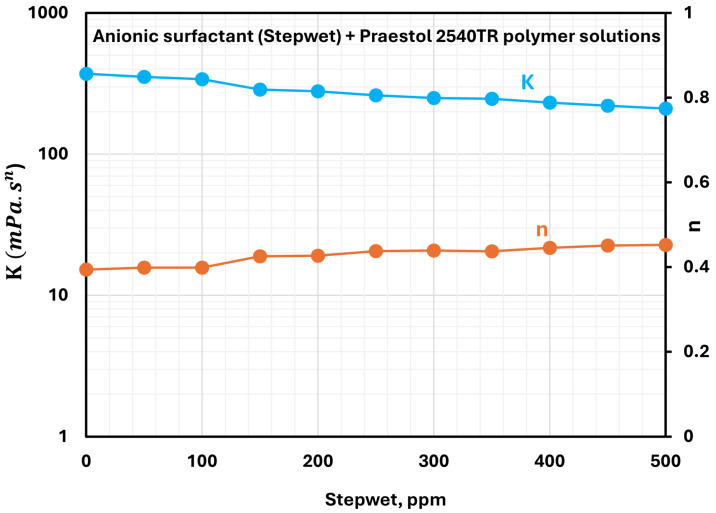
Variations of power-law parameters of anionic surfactant Stepwet + anionic Praestol 2540TR polymer solutions with surfactant concentration.

In contrast to LR-400, the consistency index of Praestol 2540TR solutions decreases with the increasing concentration of Stepwet DF-95, as shown in Figure 8. This behavior is likely due to electrostatic repulsion between the anionic polymer and the anionic surfactant, which weakens polymer chain interactions and disrupts the network structure. Despite the reduction in consistency index, all solutions retain their shear-thinning behavior, consistent with the inherent properties of Praestol 2540TR solution. The flow behavior index n increases only slightly as surfactant concentration increases.

#### 3.2.3. Cationic Surfactant (HTAB) + Polymer Solutions

**Figure 9:**
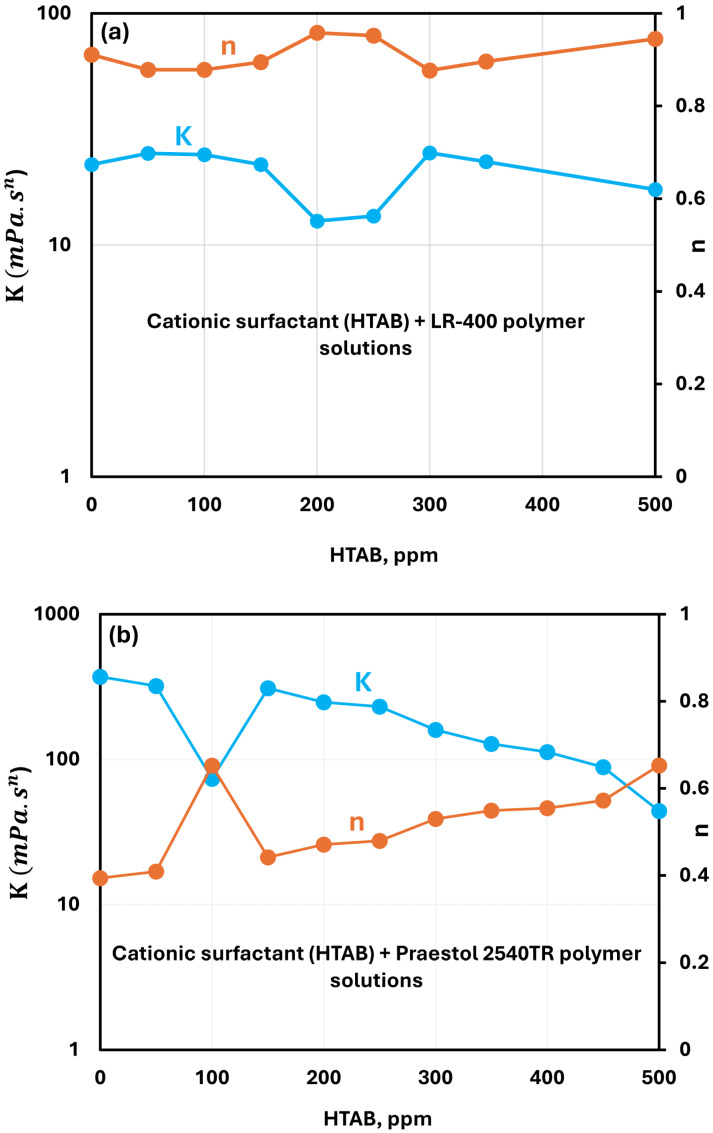
Variations of power-law parameters of cationic surfactant HTAB + polymer solutions with surfactant concentration. (**a**) HTAB + LR-400. (**b**) HTAB + Praestol 2540TR.

Figure 9a shows the effect of cationic surfactant HTAB on the rheological behavior of LR-400 solutions at a fixed polymer concentration of 5000 ppm. The consistency index K retains similar values to the pure LR-400 solution within most surfactant concentrations. However, within the 150–300 ppm range of surfactant concentration, the consistency index falls. In this range, the solution also transitions into a nearly Newtonian fluid, with flow behavior index n close to unity.

Figure 9b presents the rheological behavior of Praestol 2540TR solutions in the presence of HTAB. In general, the consistency index K decreases and the flow behavior index n increases with the addition of HTAB to Praestol 2540TR solution. However, at a surfactant concentration of 100 ppm, there is a pronounced drop in consistency index and an increase in flow behavior index. The exact cause for this observation is not clear at present.

#### 3.2.4. Zwitterionic Surfactant (Amphosol CG) + Polymer Solutions

**Figure 10:**
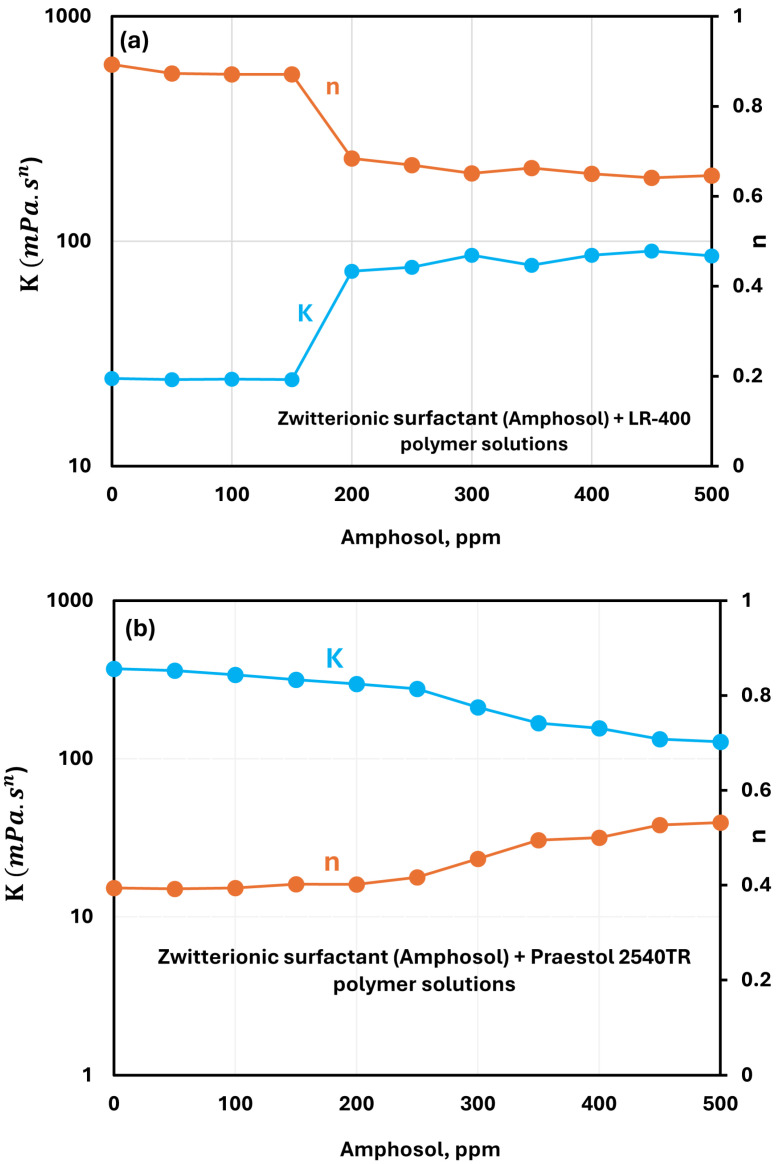
Variations of power-law parameters of cationic surfactant HTAB + polymer solutions with surfactant concentration. (**a**) Amphosol + LR-400. (**b**) Amphosol + Praestol 2540TR.

The effect of the zwitterionic surfactant Amphosol CG on the consistency index K and flow behavior index n of cationic LR-400 solutions is shown in Figure 10a. All solutions exhibit shear-thinning behavior as the flow behavior index n is less than unity. For surfactant concentrations up to 150 ppm, the power-law parameters are nearly constant. Both K and n are nearly identical to those of the pure polymer solution, reflecting minimal changes in rheological properties at low surfactant concentrations. However, at a surfactant concentration of 200 ppm, the consistency index increases noticeably and remains largely stable with further increases in surfactant concentration. This rise is likely due to the enhanced interactions between the zwitterionic surfactant and polymer chains, leading to an increase in polymer chain entaglements and the formation of more stable polymer-surfactant complexes. The solution also becomes more shear-thinning as the surfactant concentration exceeds 200 ppm.

For anionic Praestol 2540TR solutions, the addition of Amphosol CG results in a steady decrease in consistency index (see Figure 10b) when compared to the pure polymer solution. The decrease in K with the increase in surfacatnt concentration becomes stronger at surfactant concentrations larger than 250 ppm. The decrease in the consistency index is accompanied by an increase in the flow behavior index n, indicating that the polymer solution becomes less viscous and less shear-thinning with the addition of the surfactant. Thus, there occurs a reduction in the strength of the internal polymer network due to interactions between the anionic polymer and the zwitterionic surfactant.

#### 3.2.5. Nonionic Surfactant (Alfonic 1412-3 Ethoxylate) + Polymer Solutions

**Figure 11:**
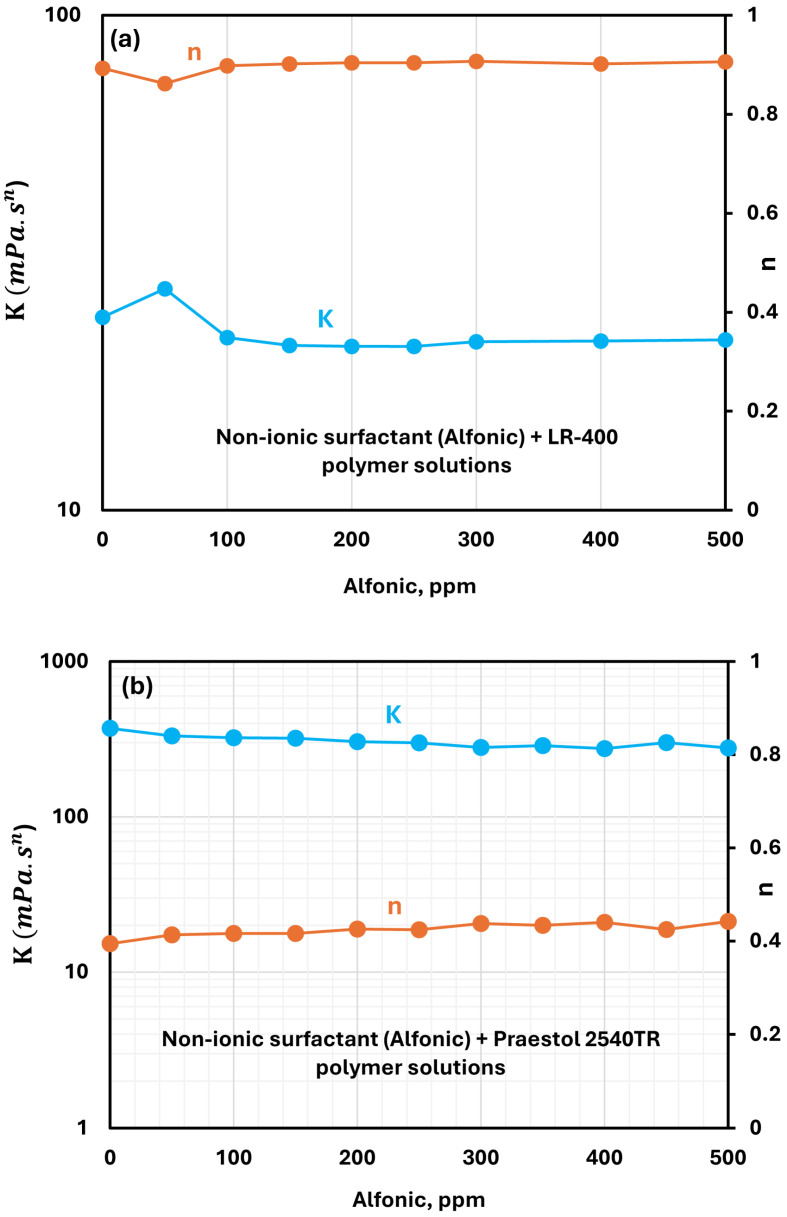
Variations of power-law parameters of non-ionic surfactant Alfonic 1412-3 ethoxylate + polymer solutions with surfactant concentration. (**a**) Alfonic + LR-400. (**b**) Alfonic + Praestol 2540TR.

The effect of the non-ionic surfactant Alfonic 1412-3 ethoxylate addition on the consistency index and flow behavior index of cationic LR-400 solutions at a fixed polymer concentration of 5000 ppm is shown in Figure 11a. Across the entire range of surfactant concentrations, the consistency and flow behavior indices remain nearly unchanged compared to the pure polymer solution, showing minimal variation. This suggests that the non-ionic surfactant does not significantly interfere with the molecular interactions responsible for the solution’s rheological properties.

The influence of Alfonic 1412-3 ethoxylate addition on the power-law parameters of anionic Praestol 2540TR solutions is shown in Figure 11b. As the surfactant concentration is increased, the flow behavior index (n) increases only slightly. At the same time, the consistency index (K) decreases slightly. Thus, surfactant-polymer interactions are weak when a non-ionic surfactant Alfonic 1412-3 ethoxylate is added to anionic Praestol 2540TR polymer solution.

#### 3.2.6. Comparisons of Rheological Behaviors of Polymer-Surfactant Solutions

**Figure 12:**
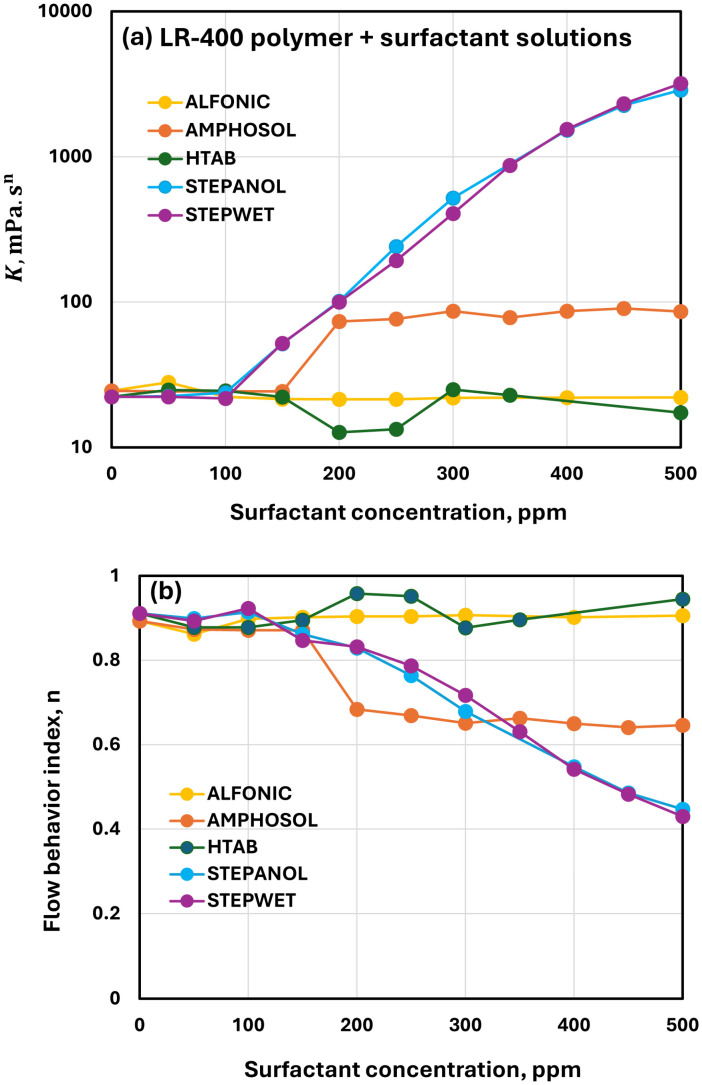
Comparison of the influence of different surfactants on the rheological properties of surfactant + cationic LR-400 solutions (LR-400 concentration fixed at 5000 ppm). (**a**) Consistency index. (**b**) Flow behavior index.

Figure 12 compares the rheological properties of solutions of different surfactants and cationic polymer LR-400 at a fixed polymer concentration of 5000 ppm. The rheological properties (consistency index K and flow behavior index n) clearly reflect strong interactions between cationic polymer and anionic surfactants (Stepanol and Stepwet) above surfactant concentrations of 100 ppm. The zwitterionic surfactant shows modest interaction above a surfactant concentration of 150 ppm. The cationic surfactant HTAB shows weak interaction in the intermediate surfactant concentration range of 150–300 ppm. The interaction between non-ionic surfactant Alfonic and cationic polymer LR-400 is negligible over the surfactant concentration range of 0–500 ppm. Thus, the strength of interaction is in the following order: Stepanol ≥ Stepwet > Amphosol > HTAB > Alfonic.

**Figure 13:**
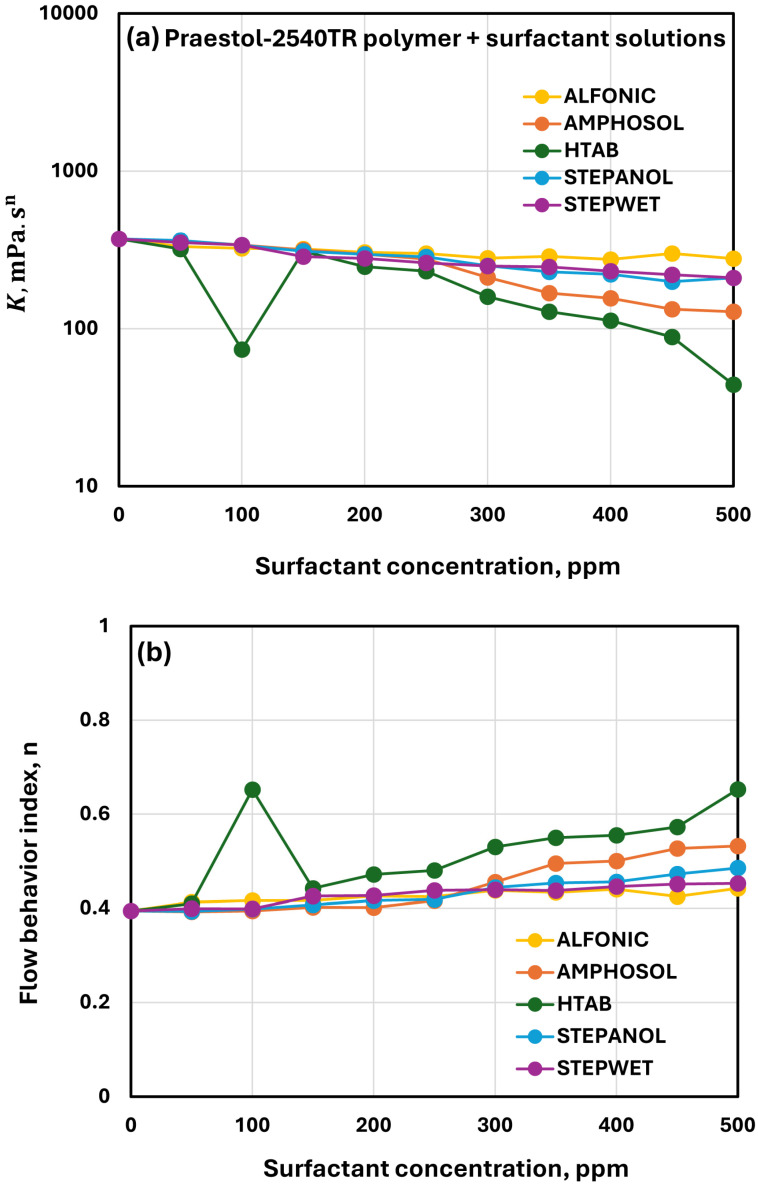
Comparison of the influence of different surfactants on the rheological properties of surfactant + anionic Praestol 2540TR solutions (Praestol 2540TR concentration fixed at 500 ppm). (**a**) Consistency index. (**b**) Flow behavior index.

Figure 13 compares the rheological properties of solutions of different surfactants and anionic polymer Praestol 2540TR at a fixed polymer concentration of 500 ppm. As compared with the cationic polymer LR-400 (see Figure 12), the effects of surfactants on the rheological properties (consistency index K and flow behavior index n) of anionic polymer Praestol 2540TR are clearly small. Furthermore, the consistency index K generally decreases and the flow behavior index increases with the addition of surfactant to the polymer solution. This clearly indicates that the addition of surfactant to anionic polymer solution disrupts the polymer chain entanglements to some extent, causing a decrease in consistency and shear-thinning. The effects of a non-ionic surfactant (Alfonic) and anionic surfactants (Stepanol and Stepwet) on the rheological properties of anionic polymer Praestol 2540TR are negligible over the full concentration range of surfactant concentration investigated (0–500 ppm). The zwitterionic surfactant (Amphosol) and the cationic surfactant (HTAB) show moderate interactions with the anionic polymer. Thus, the strength of surfactant-polymer interaction is in the following order: HTAB > Amphosol > Stepanol = Stepwet = Alfonic.

Upon comparison of Figure 12 and Figure 13, the differences in rheological behavior between cationic polymer LR-400 and anionic polymer Praestol 2540 TR are significant. Anionic surfactant-cationic polymer combinations result in high consistency and a high degree of shear-thinning, whereas anionic surfactant-anionic polymer combinations show negligible changes in rheological properties. Clearly the addition of oppositely charged anionic surfactants to the cationic polymer neutralizes the polymer chains of electric charge, resulting in their entanglements and the formation of a network of polymer chains. The entanglements of polymer chains and formation of network structure of polymer chains are expected to increase consistency and enhance the degree of shear-thinning. When the same charge anionic surfactants are added to the anionic polymer, the electrostatic repulsion between the surfactant and polymer chains hinders any changes in the entanglement and network structure of polymer chains. Consequently, the rheological properties hardly change.

### 3.3. Surface Activity of Polymer-Surfactant Solutions

#### 3.3.1. Surfactant + LR-400 Polymer Solutions

When a surfactant is added to a polymer solution, the interaction between polymer and surfactant molecules begins at the so called “critical aggregation concentration (CAC)”. The CAC is usually lower than the critical micelle concentration (CMC) of the surfactant when the surfactant and polymer molecules are oppositely charged species, that is, the interactions between the polymer and surfactant are electrostatic in nature [37,40,41,42]. However, the CAC is close to CMC when the polymer is non-ionic and the surfactant is ionic/non-ionic, resulting in hydrophobic interactions. With the increase in surfactant concentration, another critical surfactant concentration is reached when the polymer molecules become saturated with surfactant. No more surfactant can go to the polymer. This conncentration of surfactant is called the polymer saturation point (PSP) [40,41]. The CAC and PSP concentrations of surfactants are close to CMC if the surfactant-polymer interactions are weak. The surface tension and electrical conductivity plots of polymer-surfactant mixtures often show break points at surfactant concentrations corresponding to CAC and PSP points [40].

**Figure 14:**
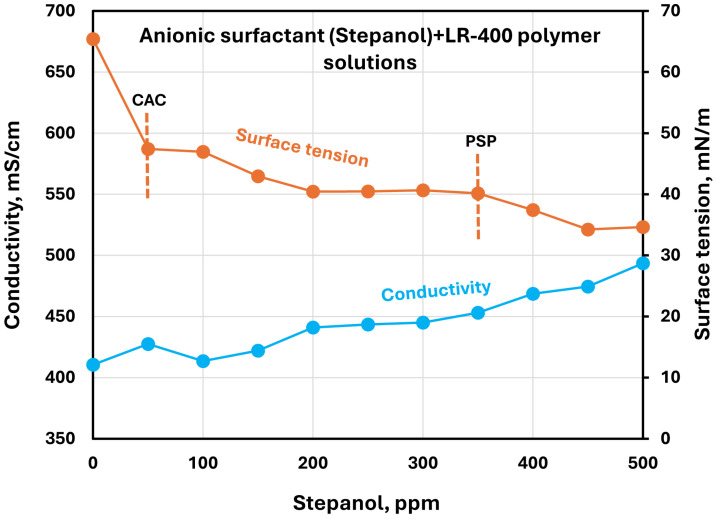
Electrical conductivity and surface tension variations with anionic surfactant (Stepanol) concentration in surfactant + cationic LR-400 polymer solutions.

The effect of anionic Stepanol WA-100 concentration on the surface tension and electrical conductivity of cationic LR-400 polymer solutions is illustrated in Figure 14. As the surfactant concentration increases, the electical conductivity increases gradually, whereas the surface tension decreases. The increase in conductivity is due to the addition of electrically charged species (surfactant molecules) to the solution. Although the conductivity and surface tension plots do not exhibit sharp enough breaks to draw definite conclusions about CAC and PSP points of surfactant-polymer interactions, they appear to be approximately as follows, as shown in the figure: CAC = 50 ppm and PSP = 350 ppm. Alternative methods such as calorimetry or spectroscopy could perhaps be used to validate these values of CAC and PSP.

**Figure 15:**
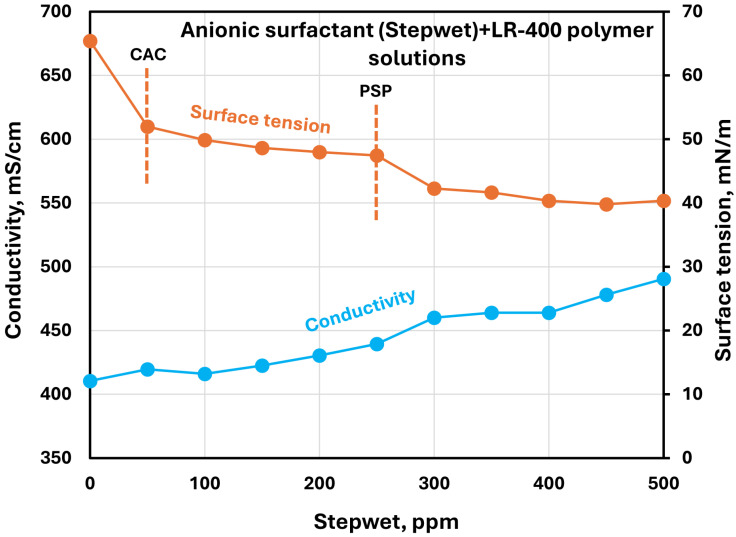
Electrical conductivity and surface tension variations with anionic surfactant (Stepwet) concentration in surfactant + cationic LR-400 polymer solutions.

**Figure 16:**
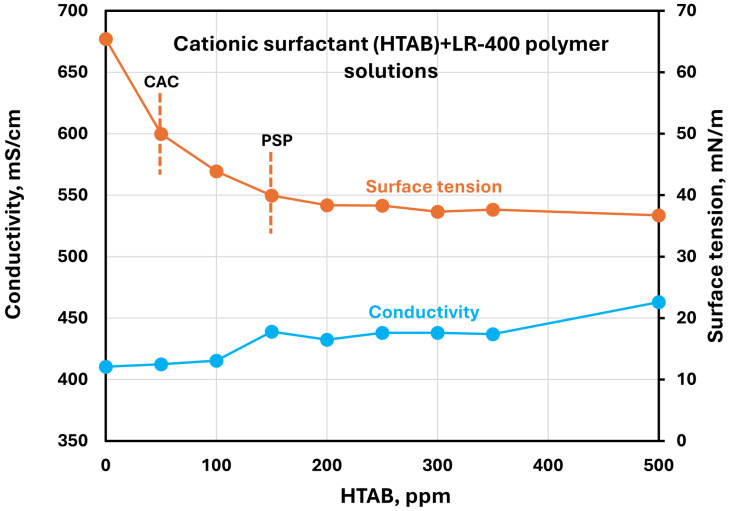
Electrical conductivity and surface tension variations with cationic surfactant (HTAB) concentration in surfactant + cationic LR-400 polymer solutions.

**Figure 17:**
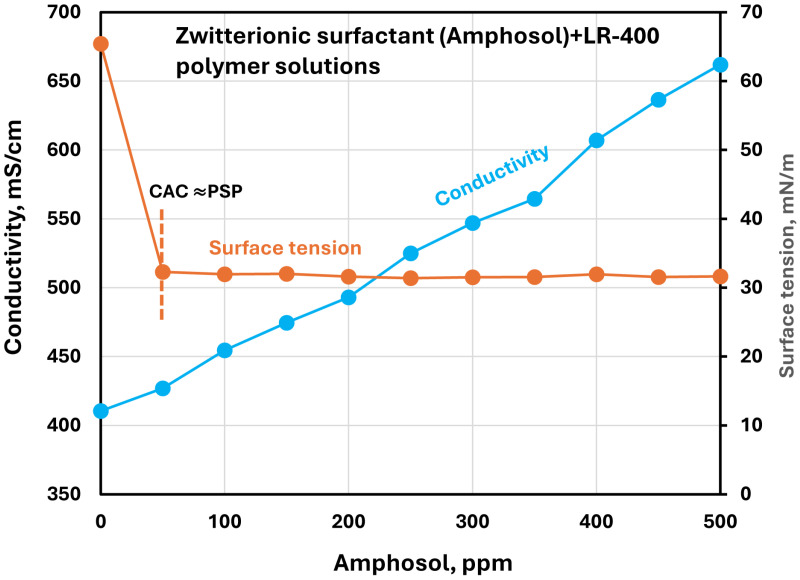
Electrical conductivity and surface tension variations with zwitterionic surfactant (Amphosol) concentration in surfactant + cationic LR-400 polymer solutions.

**Figure 18:**
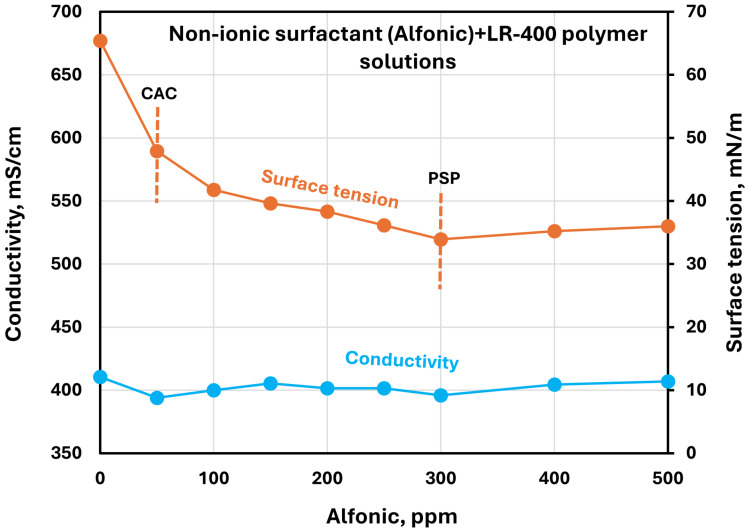
Electrical conductivity and surface tension variations with nonionic surfactant (Alfonic) concentration in surfactant + cationic LR-400 polymer solutions.

Figure 15, Figure 16, Figure 17 and Figure 18 show the electrical conductivity and surface tension plots for other surfactants (Stepwet, HTAB, Amphosol, Alfonic) in the LR-400 polymer solution. The surface tension generally decreases sharply initially with the increase in surfactant concentration but tends to level off at high surfactant concentrations. The reduction in surface tension may be interpreted in terms of adsorption of the surfactant-polymer complexes and surfactant molecules at the air-water interface, which disrupts the cohesive forces between water molecules, hence reducing surface tension. The surface tension levels out at high surfactant concentration due to saturation of the interface by surfactant-polymer complexes and surfactant molecules.

Electrical conductivity increases with the increase in ionic or zwitterionic surfactant concentrations. The changes in conductivity with surfactant addition are negligible in the case of non-ionic surfactant Alfonic. Once again, it is difficult to draw definite conclusions about the CAC and PSP points of surfactant-polymer interactions based on the conductivity and surface tension plots. They appear to be approximately as follows, as shown in the figures: Stepwet (CAC 50 ppm, PSP 250 ppm), HTAB (CAC 50 ppm, PSP 150 ppm), Amphosol (CAC ≈ PSP 50 ppm), and Alfonic (CAC 50 ppm, PSP 300 ppm).

#### 3.3.2. Comparisons of Surface Activity of Different Surfactants in LR-400 Polymer Solution

**Figure 19:**
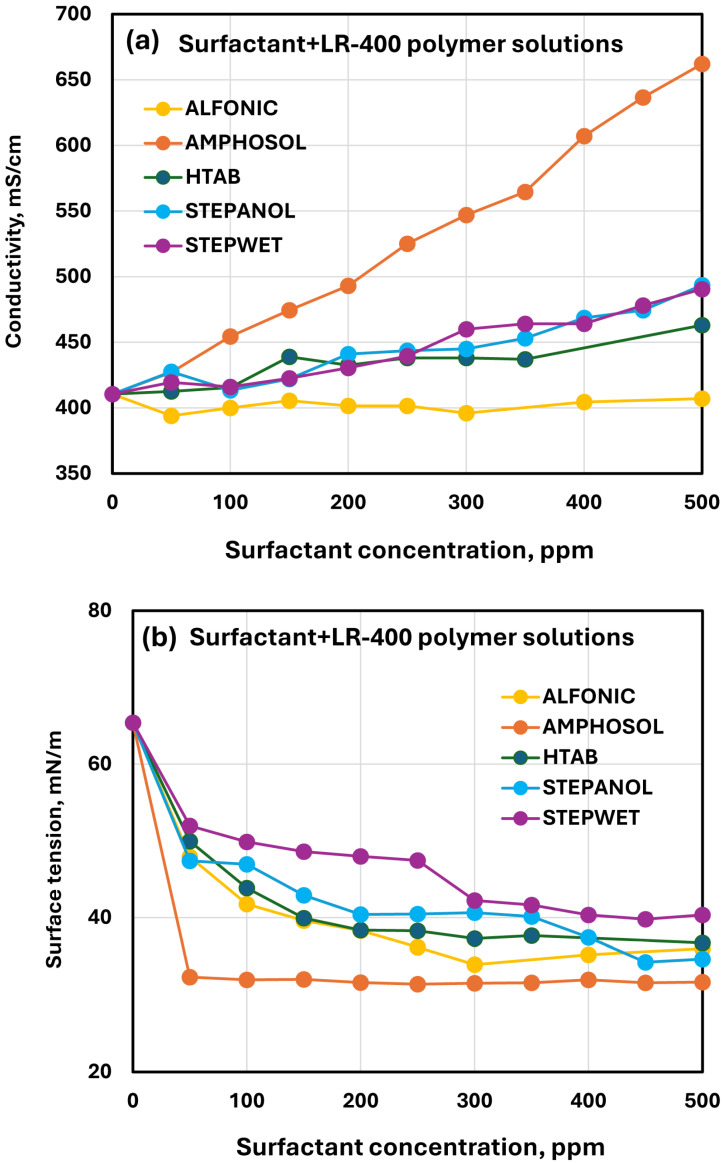
Comparisons of electrical conductivity and surface activity of different surfactants in LR-400 polymer solution. (**a**) Electrical conductivity. (**b**) Surface tension.

Figure 19 compares the conductivity and surface tension plots of different surfactants for surfactant/LR-400 polymer solutions. At any given surfactant concentration, the conductivity (see Figure 19a) increases over the pure LR-400 polymer solution in the following order: Amphosol > Stepwet ≈ Stepanol ≈ HTAB. Note that the addition of the non-ionic surfactant Alfonic to the polymer solution has no effect on the conductivity. The comparison of surface activity of different surfactants in LR-400 polymer solution is shown in Figure 19b. In all cases, the surface tension drops sharply initially and then levels off with the increase in surfactant concentration. At any given surfactant concentration, the surface tension of surfactant-polymer mixture is in the following order: Stepwet > Stepanol > HTAB > Alfonic > Amphosol. Thus, Amphosol is the most effective surfactant in reducing the surface tension.

#### 3.3.3. Surfactant + Praestol 2540TR Polymer Solutions

**Figure 20:**
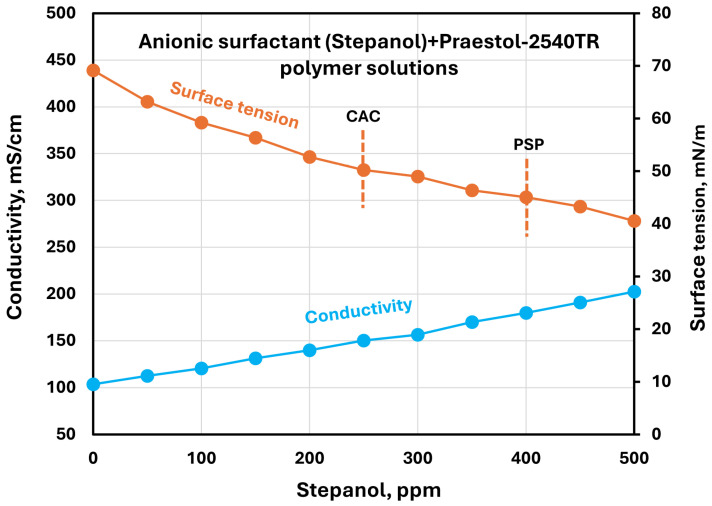
Electrical conductivity and surface tension variations with anionic surfactant (Stepanol) concentration in surfactant + anionic Praestol 2540TR polymer solutions.

**Figure 21:**
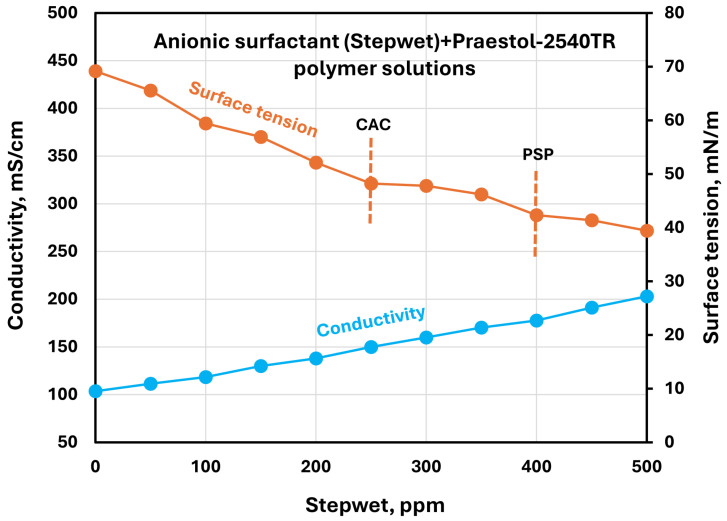
Electrical conductivity and surface tension variations with anionic surfactant (Stepwet) concentration in surfactant + anionic Praestol 2540TR polymer solutions.

**Figure 22:**
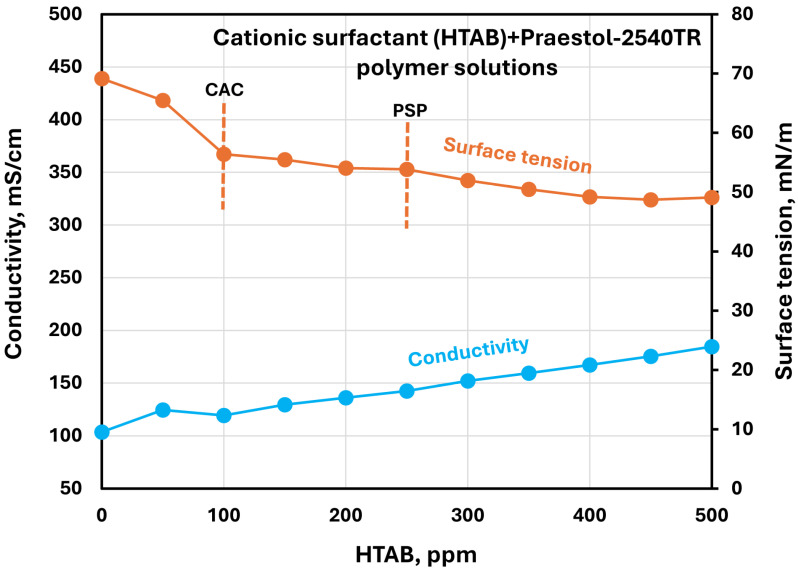
Electrical conductivity and surface tension variations with cationic surfactant (HTAB) concentration in surfactant + anionic Praestol 2540TR polymer solutions.

**Figure 23:**
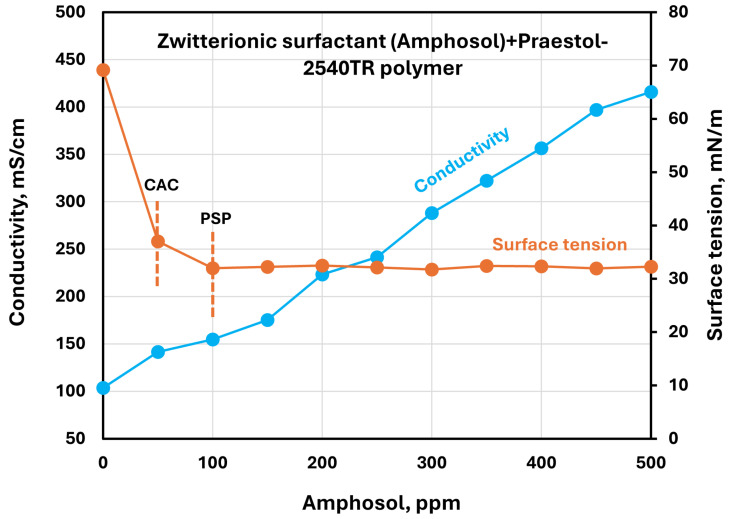
Electrical conductivity and surface tension variations with zwitterionic surfactant (Amphosol) concentration in surfactant + anionic Praestol 2540TR polymer solutions.

**Figure 24:**
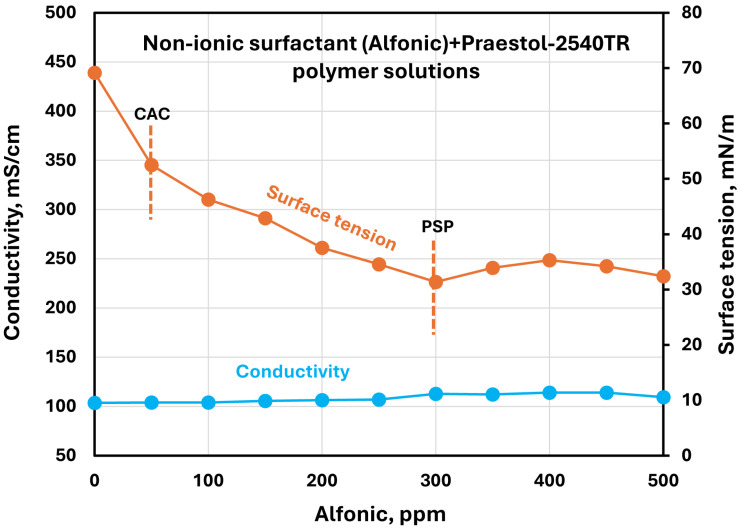
Electrical conductivity and surface tension variations with non-ionic surfactant (Alfonic) concentration in surfactant + anionic Praestol 2540TR polymer solutions.

The effect of surfactants on the surface tension and electrical conductivity of anionic Praestol 2540TR polymer solutions is shown in Figure 20, Figure 21, Figure 22, Figure 23 and Figure 24. As the surfactant concentration increases, the electrical conductivity increases gradually, whereas the surface tension decreases. Once again, the increase in conductivity is due to the addition of electrically charged species (surfactant molecules) to the solution. The changes in conductivity with surfactant addition are negligible in the case of the non-ionic surfactant Alfonic. The conductivity and surface tension plots of anionic surfactants Stepanol and Stepwet hardly exhibit any breaks, indicating negligible interactions between electrically same charged surfactants and anionic polymers. Due to repulsion between same charged surfactants and polymer molecules, the aggregation between surfactant and polymer is expected to be negligible. The CAC and PSP appear to be approximately as follows: Stepanol (CAC 250 ppm, PSP 400 ppm), Stepwet (CAC 250 ppm, PSP 400 ppm), HTAB (CAC 100 ppm, PSP 250 ppm), Amphosol (CAC 50 ppm, PSP 100 ppm), and Alfonic (CAC 50 ppm, PSP 300 ppm).

#### 3.3.4. Comparisons of Surface Activity of Different Surfactants in Praestol 2540TR Polymer Solution

**Figure 25:**
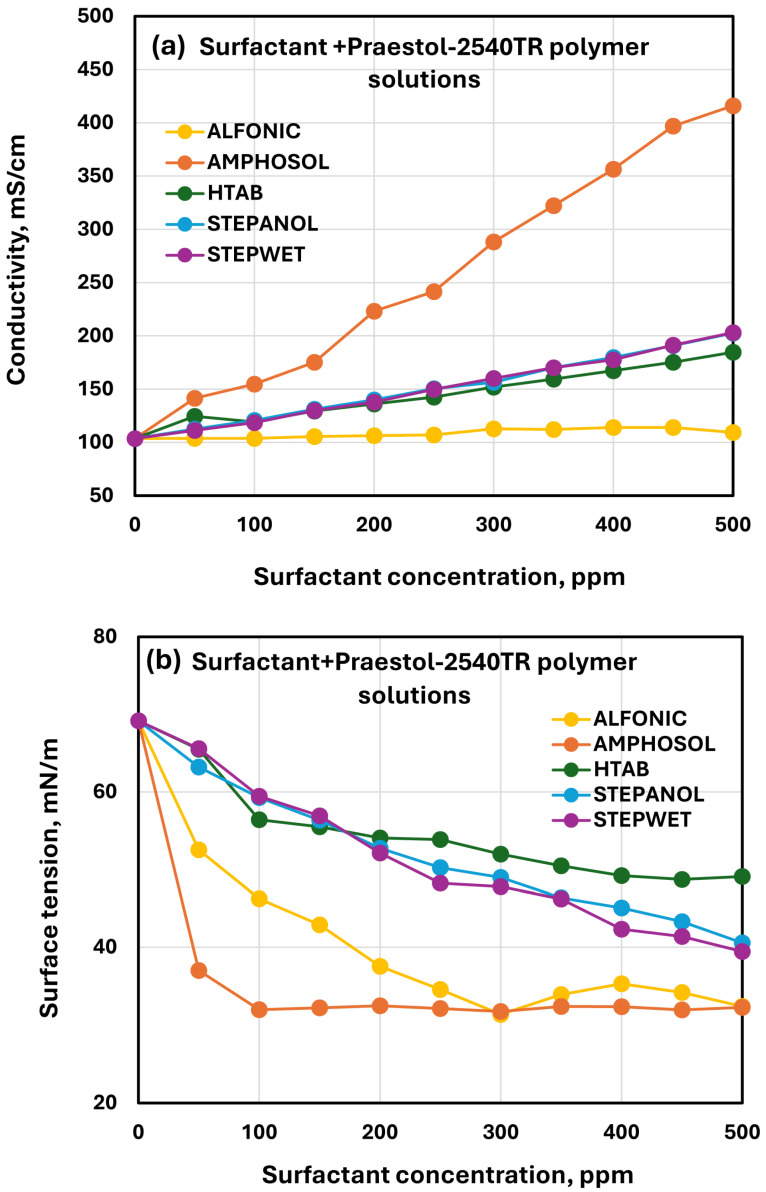
Comparisons of electrical conductivity and surface activity of different surfactants in Praestol 2540TR polymer solution. (**a**) Electrical conductivity. (**b**) Surface tension.

Figure 25 compares the conductivity and surface tension plots of different surfactants for surfactant/Praestol-2540TR polymer solutions. The electrical conductivity (see Figure 25a) increases over the pure Praestol-2540TR polymer solution at any given surfactant concentration in the following order: Amphosol > Stepwet = Stepanol ≥ HTAB. As expected, the addition of non-ionic surfactant Alfonic to polymer solution has a negligible effect on conductivity. The comparison of surface activity of different surfactants in Praestol-2540TR polymer solution is shown in Figure 25b. For zwitterionic Amphosol and non-ionic Alfonic, the surface tension drops sharply initially and then levels off with the increase in surfactant concentration. In other cases (Stepanol, Stepwet, and HTAB), the surface tension decreases with the addition of a surfactant more gradually. At any given surfactant concentration, the surface tension of surfactant-polymer mixture is in the following order: HTAB > Stepanol ≥ Stepwet > Alfonic > Amphosol. Thus, Amphosol is once again the most effective surfactant in reducing the surface tension.

## 4. Conclusions

The interactions between two polymers and five surfactants were investigated experimentally using rheology, surface tension, and conductivity measurements. The polymers studied were cationic quaternary ammonium salt of hydroxyethyl cellulose (LR-400) and anionic polyacrylamide Praestol 2540TR. The surfactants examined included anionic Stepanol WA-100, anionic Stepwet DF-95, cationic HTAB, zwitterionic Amphosol CG, and non-ionic Alfonic 1412-3 Ethoxylate. Based on the experimental results, the following conclusions can be drawn:

- The rheological behavior of cationic LR-400 polymer is strongly affected by anionic surfactants Stepanol WA-100 and Stepwet DF-95. The consistency index of solutions rises sharply, and the flow behavior index decreases substantially with the increase in surfactant concentration. Thus, solutions become more viscous and highly shear thinning with the addition of anionic surfactants to cationic LR-400 polymer. The zwitterionic surfactant Amphosol also increases the consistency of LR-400 polymer, but the increase is modest. Other surfactants have little effect on the rheological properties of LR-400 polymer solution.
- The influence of surfactants on the rheological properties of anionic Praestol 2540TR is small. Only zwitterionic Amphosol and cationic HTAB have some effect on the rheological properties at high surfactant concentrations. The consistency index decreases, and the flow behavior index increases to some extent with the increase in surfactant concentration. Other surfactants (Stepanol WA-100, Stepwet DF-95, and Alfonic 1412-3 Ethoxylate) have negligible effect on the rheological properties of polymer solution.
- The break points exhibited by surface tension and electrical conductivity plots are not sharp enough to estimate the critical aggregation concentration (CAC) and polymer saturation point (PSP) accurately. Only approximate values of CAC and PSP could be estimated in most systems.
- The results of this work offer valuable insights into tailoring polymer-surfactant systems for industrial applications, where precise control of rheological and interfacial properties is essential. The interplay between polymer charge and type, surfactant charge and type, and concentration plays a critical role in optimizing polymer-surfactant systems for industrial applications. Strong electrostatically attractive interactions between oppositely charged anionic surfactants and cationic polymer enhance rheological properties in LR-400 solutions due to charge neutralization and entanglements of polymer chains, while electrostatically repulsive interactions between anionic surfactant and anionic Praestol 2540TR system result in minor changes in rheological properties. Understanding these relationships is essential for designing fluids with precise rheological and surface activity characteristics for applications such as enhanced oil recovery, hydraulic fracturing and drilling.

## Figures and Tables

**Table 1:** Different surfactants investigated in this work [37].

Commercial Name	Chemical Name	Type of Surfactant
HTAB	Hexadecyltrimethyl ammonium bromide	Cationic
Alfonic 1412-3 Ethoxylate	Ethoxylated alcohols	Non-ionic
Stepwet DF-95	Sodium Lauryl Sulfate based surfactant	Anionic
Stepanol WA-100	Sodium Lauryl Sulfate based surfactant	Anionic
Amphosol CG	Cocamidopropyl Betaine	Zwitterionic

## Data Availability

The original contributions presented in this study are included in the article. Further inquiries can be directed to the corresponding author.
